# Rexinoid NEt-3IB Promotes Resident Macrophage Gene Expression and Mitigates Desiccation-Induced Ocular Surface Disease

**DOI:** 10.1167/iovs.67.4.31

**Published:** 2026-04-14

**Authors:** Jehan Alam, Yangluowa Qu, Jianming Shao, Ebru Yaman, Karen Zheng, Hiroki Kakuta, Stephen C. Pflugfelder

**Affiliations:** 1 Ocular Surface Center, Department of Ophthalmology, Baylor College of Medicine, Houston, Texas, United States; 2 Laboratory of Bioorganic and Medicinal Chemistry, Okayama University Graduate School of Medicine, Dentistry and Pharmaceutical Sciences, Okayama, Japan

**Keywords:** single-cell RNA sequencing, NEt-3IB, goblet cells, desiccation stress

## Abstract

**Purpose:**

To evaluate the effects of rexinoid NEt-3IB on desiccating stress–induced dry eye, as well as monocyte/macrophage gene expression and cellular trajectory.

**Methods:**

Eyes were topically treated with rexinoid NEt-3IB (5 µM) or vehicle three times a day for 5 days of desiccating stress–induced dry eye. Single-cell RNA sequencing (RNA-seq) profiled gene expression in conjunctival immune cells. RNA-seq was also used to evaluate gene expression in lipopolysaccharide (LPS)-stimulated, dexamethasone-treated (Dex, 1 µM), or NEt-3IB–treated (1–1000 nM) cultured monocytes. Cellular state trajectory and latent time were inferred with scVelo, and latent-time-associated genes were identified using Monocle 3. Permeability to Oregon Green–labeled dextran was used to assess corneal barrier, and conjunctival goblet cell parameters were measured in periodic acid–Schiff (PAS)-stained wholemounts.

**Results:**

NEt-3IB significantly stimulated homeostatic, phagocytotic, and anti-inflammatory gene expression and suppressed inflammatory gene signatures in conjunctival monocyte/macrophage cells compared to vehicle (*P*_adj_ < 0.05). Latent time trajectory analysis and flow cytometry further revealed that NEt-3IB preserved resident macrophage-associated gene and protein expression. Compared to vehicle, corneal labeled dextran uptake was lower (*P* = 0.001) and conjunctival goblet cell total (*P* = 0.001) and single-cell (*P* = 0.01) areas were higher in the NEt-3IB group. The impact of NEt-3IB on differentially expressed genes in cultured monocytes mirrored its effects in vivo. NEt-3IB and dexamethasone suppressed inflammatory mediator expression; however, NEt-3IB enhanced the expression of homeostatic factors, including *Igf1* and *Il10*.

**Conclusions:**

Rexinoid NEt-3IB suppresses inflammatory and stimulates homeostatic gene expression in the monocyte/macrophage lineage and desiccation-induced ocular surface disease. Rexinoid therapy may prove to be a novel approach to treat dry eye by stimulating endogenous production of homeostatic/anti-inflammatory factors.

Resident macrophages are long-lived, tissue-resident immune cells that maintain homeostasis, suppress inflammation, and promote tissue repair after injury.^1^ These cells originate from yolk sac progenitors during embryogenesis and from circulating monocytes recruited after birth.^2^ The nuclear receptor retinoid X receptor alpha (RXRα) programs monocytes toward resident macrophages by regulating the expression of genes involved in phagocytosis and immune homeostasis.^3^ We previously reported that the Pinkie RXR⍺ mutant mouse strain that develops spontaneous dry eye has a 20% reduction in conjunctival resident macrophages and a significant reduction in expression of the homeostatic cytokine *IGF1* by these cells.^4^ We found that desiccating stress–induced dry eye promotes recruitment of C-C chemokine receptor type 2 positive (CCR2⁺) monocytes from the circulation and upregulates their expression of inflammatory cytokines, chemokines, and other mediators associated with dry eye.^5^^,^^6^ Desiccating stress can also stimulate expression of anti-inflammatory, phagocytic, and homeostatic genes in CCR2⁻ resident macrophages.^6^ These resident macrophages appear to mitigate ocular surface inflammation, as their depletion before initiating desiccating stress exacerbates conjunctival inflammation and goblet cell loss. Notably, RXRα pathway activation was found to be higher in conjunctival resident macrophages than in CCR2⁺ inflammatory monocytes and macrophages.^6^ Collectively, these findings suggest that pharmacological activation of RXRα could enhance resident macrophages function, suppress inflammation, and improve ocular surface disease in experimental dry eye.

RXRα agonists, termed rexinoids, include natural molecules such as 9-*cis*-retinoic acid, honokiol (a compound found in magnolia bark), and various dietary and endogenous lipids, such as docosahexaenoic acid (DHA) from fish oil, oleic acid, and arachidonic acid.^7^ Because these molecules are highly lipophilic, their poor water solubility makes it difficult to formulate them into aqueous eye drops, which are generally better tolerated than oil-based formulations.

The purpose of this study was to test the hypothesis that synthetic rexinoid NEt-3IB sodium salt (hereafter, NEt-3IB) modulates desiccating stress–induced ocular surface monocyte/macrophage gene expression and the severity of ocular surface epithelial disease. During short-term desiccating stress, NEt-3IB was found to maintain ocular surface resident macrophage gene expression and suppress development of desiccating stress–induced corneal and conjunctival disease.

## Materials and Methods

### Animal Protocol and Care

All animal experiments complied with the ARVO Statement for the Use of Animals in Ophthalmic and Vision Research and were reviewed and approved by the Institutional Animal Care and Use Committee at Baylor College of Medicine (BCM). Female C57BL/6J (B6) mice were obtained from The Jackson Laboratory (Bar Harbor, ME, USA) and used at 10 to 12 weeks of age. Animals were maintained under standard housing conditions with controlled temperature (22°–24°C), relative humidity (50%–75%), and a 12-hour light/dark cycle. Food and water were available ad libitum. Mice were acclimatized for 2 weeks prior to experimental procedures.

### Desiccating Stress Model and Experimental Design

Animals were randomly assigned to four experimental groups: (1) control (non-stressed [NS]), (2) desiccating stress for 5 days without treatment (DS5), (3) desiccating stress for 5 days plus vehicle (DS5+vehicle; topical saline drops) three times per day, and (4) desiccating stress for 5 days plus 5-µM NEt-3IB (DS5+NEt-3IB) three times per day. Mice in the non-stressed group were housed under standard conditions with 50% to 75% relative humidity and a temperature of 20°C to 22°C. Animals in the other three groups were maintained in an environmentally controlled chamber (Darwin Chambers, St. Louis, MO, USA) with relative humidity maintained at 20% to 25% and temperature at 22°C to 24°C. Desiccating stress was induced as previously described.^8^ Briefly, tear secretion was inhibited by administering scopolamine hydrobromide (0.5 mg/mL; Greenpark Compounding Pharmacy, Houston, TX, USA) in drinking water. In addition, mice were housed in cages with one perforated plastic side panel exposed to constant airflow (50 ft^3^/min) from a 140-mm blade computer fan positioned approximately 6 inches away for 16 hours per day over 5 consecutive days. During the stress period, mice in the treatment groups received either vehicle (saline) or NEt-3IB (5 µM) topically applied to both eyes three times daily. Synthesis of NEt-3IB sodium salt is described in the Supplementary Methods.

### Conjunctival Tissue Digestion, Flow Cytometry, and Immune Cell Sorting

Conjunctival single-cell suspensions were prepared as previously described.^9^ Briefly, the bulbar conjunctiva (epithelium and substantia propria) was dissected from the limbus to its reflection onto the upper and lower tarsal conjunctiva, finely minced, and digested with 0.1% type IV collagenase for 1 hour. For immune cell isolation, Fc receptors were blocked by incubating the samples with anti-CD16/32 (5 minutes at room temperature) followed by staining with anti-CD45. An infrared fluorescent viability dye was used to exclude dead cells. Lymphocytes were identified by forward scatter area (FSC-A) versus side scatter area (SSC-A), doublets were excluded using FSC-A versus forward scatter height (FSC-H) and SSC-A versus side scatter height (SSC-H) plots, and live cells were selected with the viability dye. CD45⁺ cells for sequencing were sorted using a BD FACSAria II Flow Cytometer (BD Biosciences, Franklin Lakes, NJ, USA) at the BCM Cytometry and Cell Sorting Core.

Conjunctival single-cell suspensions were used for surface and intracellular flow cytometric analysis. Following tissue digestion, cells were incubated with GolgiPlug 1 µg/mL (555029; BD Pharmingen, San Diego, CA, USA) for 6 hours to allow intracellular protein retention before antibody staining. After the incubation, cells were washed and first incubated with anti-CD16/32 Fc block antibody (clone 2.4G2, 553141; BD Pharmingen) for 5 minutes at room temperature to reduce nonspecific binding. Cells were then stained with the following surface antibodies: BV510 anti-CD45 (clone 30-F11, 103138; BioLegend, San Diego, CA, USA), PE-Cy7 anti-CD11b (clone M1/70, 552850; BD Pharmingen), Alexa Fluor 647 anti-MRC1 (clone Y17-505, 568808; BD Pharmingen), and PerCP/Cyanine5.5 anti-mouse CX3CR1 (clone SA011F11, 149010; BioLegend). Live/dead discrimination was performed using LIVE/DEAD Fixable Near-IR Dead Cell Stain Kit (L10119; Thermo Fisher Scientific, Waltham, MA, USA). Following surface staining, cells were pelleted, residual staining buffer was removed, and the cell pellet was gently resuspended. Samples were then fixed and permeabilized with 1× Fix/Perm buffer (562574; BD Pharmingen) for 1 hour at 2°C to 8°C. After fixation/permeabilization, cells were washed with 1× Perm/Wash buffer and incubated with Alexa Fluor 488 anti-IGF-1 antibody (clone H-9, sc-518040; Santa Cruz Biotechnology, Dallas, TX, USA) diluted in 1× Perm/Wash buffer for intracellular staining. Flow cytometric analysis was performed by first identifying cells based on forward- and side-scatter properties (FSC-A vs. SSC-A), followed by doublet exclusion using FSC-A versus forward scatter width (FSC-W) and SSC-A versus side scatter width (SSC-W). Dead cells were excluded using the Near-IR viability dye. Immune cells were identified as CD45^+^ cells, and myeloid cells were further analyzed as CD45^+^CD11b^+^ populations for expression of CX3C motif chemokine receptor 1 (CX3CR1), mannose receptor C-type 1 (MRC1), and intracellular insulin-like growth factor 1 (IGF-1).

### Library Preparation and Sequencing

Single-cell transcriptome libraries were prepared using the Chromium Next GEM Single Cell 3′ Reagent Kit v3.1 (10X Genomics, Pleasanton, CA, USA) at the BCM Single Cell Genomics Core, as previously described.^8^^–^^10^ Briefly, single cells were combined with reverse transcription reagents, Gel Beads containing barcoded oligonucleotides, and oil on a Chromium Controller (10X Genomics) to generate Gel Beads-in-Emulsions (GEMs). Within each GEM, full-length cDNA was synthesized and uniquely barcoded for its cell of origin. Following GEM disruption, the cDNA from all cells was pooled and purified with Dynabeads MyOne Silane beads (37002D; Thermo Fisher Scientific), then subjected to polymerase chain reaction (PCR) amplification. The amplified cDNA underwent enzymatic fragmentation to achieve the optimal size range, end repair, A-tailing, and adapter ligation, followed by a final round of amplification to generate sequencing-ready libraries. Library quality was assessed using a Thermo Scientific NanoDrop spectrophotometer and Agilent 2100 Bioanalyzer (Agilent Technologies, Santa Clara, CA, USA). Libraries were quantified by quantitative PCR with the KAPA Illumina/Universal Library Quantification Kit (KK4824; Illumina, San Diego, CA, USA) and sequenced on the Illumina NovaSeq 6000 platform.

### Bioinformatic Analysis of Single-Cell RNA Sequencing Data

Raw sequencing reads in FASTQ format were processed using the 10X Genomics Cell Ranger Count pipeline (v8.0.0; https://cloud.10xgenomics.com) against the mouse reference genome (GRCm39, 2024-A), which performed alignment, barcode assignment, and unique molecular identifier (UMI) quantification with default parameters. Spliced and unspliced reads were quantified from Cell Ranger–generated BAM files using velocyto 0.17.17 with default parameters. Ambient RNA contamination was corrected with SoupX, then quality control filters were applied to exclude cells with fewer than 300 detected features, fewer than 500 unique molecular identifiers, and those with >5% mitochondrial gene content. Lowly expressed genes detected in fewer than 10 cells were also excluded. Doublet detection was carried out using scDblFinder, and only singlet cells were retained. Datasets from the four experimental groups (NS, DS5, DS5+vehicle, and DS5+NEt-3IB) were then concatenated using the anndata.AnnData.concatenate function. Highly variable genes (HVGs) were identified using the scanpy.pp.highly_variable_genes function, and the top 10,000 HVGs was retained for batch correction and integrated using scVI 1.2.0^11^ with parameters *n*_layers = 2 and *n*_latent = 50. The resulting latent representation was used to construct a nearest-neighbor distance matrix, from which cell clusters were identified using the Leiden algorithm. Two-dimensional visualization was generated via Uniform Manifold Approximation and Projection (UMAP). Cluster-specific marker genes were identified using the scanpy.tl.rank_genes_groups function. Clusters enriched for epithelial or stromal markers (Krt12, Tjp1, Aldh3a1, Epcam, Fxyd3, Clrn1, Htr1d, Grip1, and Zp4) were excluded from downstream analysis, as previously described.^10^

### Trajectory Inference of Monocyte and Macrophage Lineage Cells

Trajectory of monocyte and macrophage lineage cells was inferred using RNA velocity. First, the subset of monocyte/macrophage lineage cells was isolated and integrated following the same single-cell integration workflow described in the section above. Subsequent RNA velocity analyses were performed by Scanpy and scVelo 0.3.2.^12^ Gene selection and normalization were performed using the scvelo.pp.filter_and_normalize function. We filtered out genes detected in fewer than 20 cells in both spliced and unspliced matrices, and the top 10,000 HVGs were then retained for RNA velocity estimation. Principal component analysis was applied to the normalized expression matrix, and a *k*-nearest neighbor (kNN) graph (*k* = 30) was computed using the top 50 principal components. Cell–cell moments were estimated with scvelo.pp.moments using 30 neighbors. RNA velocity vectors were inferred with the scvelo.tl.velocity function in dynamical mode, and the corresponding latent time was estimated using scvelo.tl.latent time. Genes associated with latent times were identified using Monocle 3 by fitting a negative binomial regression model.^13^^,^^14^ Genes were considered significantly different if they were expressed in more than 100 cells and had a false discovery rate–adjusted *q* value ≤ 0.05.

### Assessment of Corneal Barrier Function

Corneal epithelial barrier integrity was evaluated using 70-kDa Oregon Green–conjugated dextran (Invitrogen, Carlsbad, CA, USA), following established protocols.^15^ Briefly, 1 µL of Oregon Green dextran (50 mg/mL) was applied to the ocular surface 1 minute prior to euthanasia. Eyes were then rinsed with 2 mL phosphate-buffered saline (PBS) directed from both the temporal and nasal sides. Fluorescent images were captured using a high-resolution digital camera (CoolSNAP HQ2; PhotoMetrics, Tucson, AZ, USA) mounted on a stereoscopic zoom microscope (SMZ1500; Nikon, Tokyo, Japan) with excitation at 470 nm. The extent of Oregon Green dextran uptake was quantified from digital images using NIS Elements 3.0 (Nikon). A 2-mm-diameter circular region of interest was centered on the cornea, and fluorescence intensity was software measured within this zone by two masked observers. The mean fluorescence values for each group were used for statistical analysis.

### Conjunctiva–Cornea Wholemount Periodic Acid Schiff Staining

Conjunctiva–cornea wholemount preparation was performed following the method described by Shi et al.^16^ Briefly, the entire eye, including the globe, eyelid, and associated tissues, was excised and immediately fixed in 4% paraformaldehyde (PFA) for 15 minutes at 4°C. After fixation, excess skin and fascia were carefully removed, and a posterior-to-anterior incision was made. The lens, scleral tissue, and iris were dissected away while maintaining the integrity of the cornea and conjunctiva and avoiding epithelial injury. The conjunctiva was then bisected, each half retaining a portion of the cornea. To flatten the tissue, a radial cut was made from the corneal center to the limbus. The conjunctiva–cornea wholemounts were then transferred to 4% PFA and fixed overnight at 4°C. Following fixation, meibomian glands were removed, and the conjunctival stroma was thinned as much as possible to improve staining and visualization. Wholemounts were placed in a 96-well plate for periodic acid–Schiff (PAS) staining. Samples were immersed in 0.5% periodic acid for 2 minutes, rinsed 10 times in 1× PBS, and incubated in Schiff's reagent for 2 minutes, followed by another 10 rinses in 1× PBS. Tissues were flattened on slides and then examined and imaged at 2× magnification for measuring PAS^+^ area and 10× for measuring single-cell areas using a Nikon light microscope. For quantitative analysis, the conjunctiva was divided into three anatomical regions according to Shi et al.^16^: palpebral (∼20%, covering the meibomian glands), fornix (∼50%, folded portion), and bulbar (∼30%, adjacent to the cornea). The percentage of the area in each region covered by PAS^+^ cells was measured using ImageJ and NIS Elements software. Mean cell area was calculated by summing the areas of 1000 randomly selected PAS^+^ cells (*S*_1000_) in the fornix region with ImageJ and dividing by 1000: mean cell area (µm^2^/cell) = (*S*_1000_/1000).

### Immunofluorescence, Lectin Labeling, and Confocal Imaging

Upper and lower conjunctival tissues taken from the bulbar and forniceal regions of both eyes were carefully dissected under a stereomicroscope and immediately rinsed in PBS. Samples were fixed in cold 100% methanol for 15 minutes at −20°C, followed by a 10-minute PBS wash. Permeabilization was performed with PBS containing 0.3% Triton X-100 and 0.1% Tween 20 for three cycles of 10 minutes each at room temperature with gentle agitation. After blocking with 20% goat serum at room temperature, tissues were incubated in wheat germ agglutinin (WGA) conjugated to Alexa Fluor 488 for 1 hour in the dark with mild shaking. Excess lectin was removed by washing with PBS containing 0.3% Triton X-100 (three times for 5 minutes each), followed by an additional 10-minute PBS rinse. Nuclei were counterstained with Hoechst 33342 for 1 hour at room temperature, then washed again in PBS for 10 minutes. The stained tissues were mounted on glass slides, flattened under coverslips, and imaged using a Nikon A1 scanning confocal microscope with 0.8-µm *Z*-step intervals. Goblet measurements were performed with NIS Elements software. Cell number and fluorescence intensity were measured across six or seven fields randomly selected from the upper and lower conjunctiva at 40× magnification. Additionally, the volume of WGA-positive glycoproteins in goblet cells was measured in three-dimensional (3D) reconstructions generated from 70 to 80 *z*-stack images from each of six or seven distinct fields per sample.

For conjunctival samples, tissues were incubated with anti–IGF-1R antibody and WGA diluted in blocking buffer containing 5% serum. For corneal samples, tissues were incubated with anti–IGF-1R and βIII-tubulin antibodies diluted in blocking buffer containing 5% serum. The following primary antibodies were used: βIII-tubulin (ab215037; Abcam, Cambridge, UK) and human/mouse IGF-1R (AF-305-NA; R&D Systems, Minneapolis, MN, USA). After overnight incubation, tissues were washed three times for 5 minutes each in washing buffer with gentle shaking at room temperature. Samples were then incubated with the appropriate secondary antibodies diluted in blocking buffer for 2 hours at room temperature with gentle shaking. After washing, both conjunctival and corneal tissues were counterstained with 4′,6-diamidino-2-phenylindole (DAPI) for nuclear visualization. Samples were then washed three times for 5 minutes each, mounted on slides, and flattened with coverslips.

### Monocyte Adoptive Transfer

Bone marrow–derived cells were obtained from C57BL/6 (B6) mice or from the Pinkie strain carrying an RXRα mutation.^4^ The B6-derived cultures were maintained in the presence or absence of the RXRα antagonist HX531 (1 µM; Tocris Bioscience, Bristol, UK) for 3 days. Following culture, monocytes were purified using a monocyte isolation kit according to the manufacturer's instructions (Miltenyi Biotec, Bergisch Gladbach, Germany). A total of 2 × 10^6^ purified monocytes from each condition (B6 ± HX531 or Pinkie) were injected into the inferior bulbar conjunctiva of RAG1KO recipient mice. On the following day (4 days after culture initiation), mice were subjected to 3 days of the desiccating stress dry eye protocol described above, after which ocular tissues were collected for goblet cell analysis.

### Monocyte In Vitro Stimulation and Bulk RNA Sequencing

Bone marrow–derived cells were seeded at a density of 2 × 10^7^ cells per 100-mm culture dish in 10 mL of complete medium composed of Gibco RPMI 1640 medium supplemented with 10% heat-inactivated fetal calf serum, 50-µg/mL gentamicin, and 1.25-µg/mL amphotericin B (all, Thermo Fisher Scientific) in the presence of recombinant granulocyte–macrophage colony-stimulating factor (GM-CSF; 20 ng/mL; PeproTech, Cranbury, NJ, USA). After 3 days of culture, monocytes were purified using a monocyte isolation kit (Miltenyi Biotec) following the manufacturer's instructions. Purified monocytes (5 × 10^5^ cells/well) were plated in 48-well plates and pretreated for 1 hour with dexamethasone (1 µM) or increasing concentrations of NEt-3IB (10, 100, or 1000 nM). Cells were subsequently stimulated with lipopolysaccharide (LPS; 0.5 µg/mL) for 4 hours, after which total RNA was extracted using the RNeasy Plus Mini Kit (74134; QIAGEN, Venlo, The Netherlands) according to the supplier's protocol. The concentration and purity of RNA were assessed using a NanoDrop 1000 spectrophotometer. Messenger RNA was enriched using poly-T oligo-attached magnetic beads. First-strand cDNA was synthesized with random hexamers, followed by second-strand synthesis using deoxythymidine triphosphate. Libraries were ready after end repair, A-tailing, adapter ligation, size selection, amplification, and purification. Library quality and concentration were assessed using Qubit, real-time PCR, and Agilent Bioanalyzer. Sequencing was performed on Illumina platforms according to library concentration and data requirements. Raw reads were processed with fastp software to remove adapters, low-quality reads, and poly-N sequences. Clean reads were mapped to the reference genome using Hisat2 2.0.5, and gene-level counts were obtained with FeatureCounts 1.5.0-p3. Gene expression was quantified as fragments per kilobase million (FPKM). Differential expression analysis was performed using DESeq2, applying an adjusted *P* ≤ 0.05 and absolute fold change ≥ 2.

### Cytokine Quantification by ELISA

The monocyte culture and stimulation protocol was identical to that used for RNA sequencing (see above), except that LPS-stimulated cells were incubated for 20 hours instead of 4 hours. Supernatants were harvested, and cytokine (IL-1β, IGF-1, IL-10) concentrations were measured using R&D Systems DuoSet ELISA Kits according to the manufacturer's instructions.

### Data Availability

All raw sequencing data reported have been deposited at this accession URL: https://singlecell.broadinstitute.org/single_cell/study/SCP3406. All code and scripts related to the single-cell RNA (scRNA) sequencing computational analysis of this study are publicly accessible via the GitHub repository: https://github.com/jshao-hou/dryeye_immune_scRNA.

## Results

### NEt-3IB Maintains Resident Macrophage Gene Expression Profile

We found that macrophages are the most abundant immune cell population in the mouse conjunctiva.^6^ Macrophages can be classified as CCR2^+^ monocyte derived or CCR2^–^ tissue resident macrophages that express *Timd4*, *Lyve1*, and *Folr2* (referred to as TLF^+^).^2^ CCR2^+^ monocytes are recruited from the blood to the conjunctiva in response to desiccating stress.^5^ These cells polarize to inflammatory or homeostatic resident macrophages depending on the local signals they receive. We found the nuclear receptor RXR⍺ and its heterodimeric partner peroxisome proliferator-activated receptor gamma (PPARγ) are among the most activated pathways in resident macrophages and that the RXR⍺ agonist NEt-3IB increases expression of the resident macrophage marker *Mrc1* (CD206) by cultured bone marrow monocytes.^6^ Based on these findings, we compared the effects of topically applied NEt-3IB on conjunctival immune cell gene and protein expression and severity of desiccation-induced ocular surface disease.

The UMAP and percentages of conjunctival immune cells identified by scRNA sequencing are shown in Supplementary Figure S1A. TLF^+^CCR2^–^ and TLF^–^CCR2^+^ monocyte/macrophages cells (Supplementary Fig. S1A) comprise >60% of all conjunctival immune cells. These two groups of monocyte/macrophage cells have distinct gene expression patterns (Supplementary Fig. S2). The greatest treatment-induced changes in gene expression were observed in these cells, and based on this finding we performed trajectory analysis to model the pseudo-temporal progression of the monocyte/macrophage lineage cell population across the four experimental groups: NS, DS5, DS5+vehicle, and DS5+NEt-3IB. Using Monocle 3, we identified 2430 genes significantly positively correlated with latent time and 1905 genes significantly negatively correlated (*q* ≤ 0.05) (listed in Supplementary Table S1). DEGs in the other immune cell populations are reported in Supplementary Table S2.

**Figure 1.**
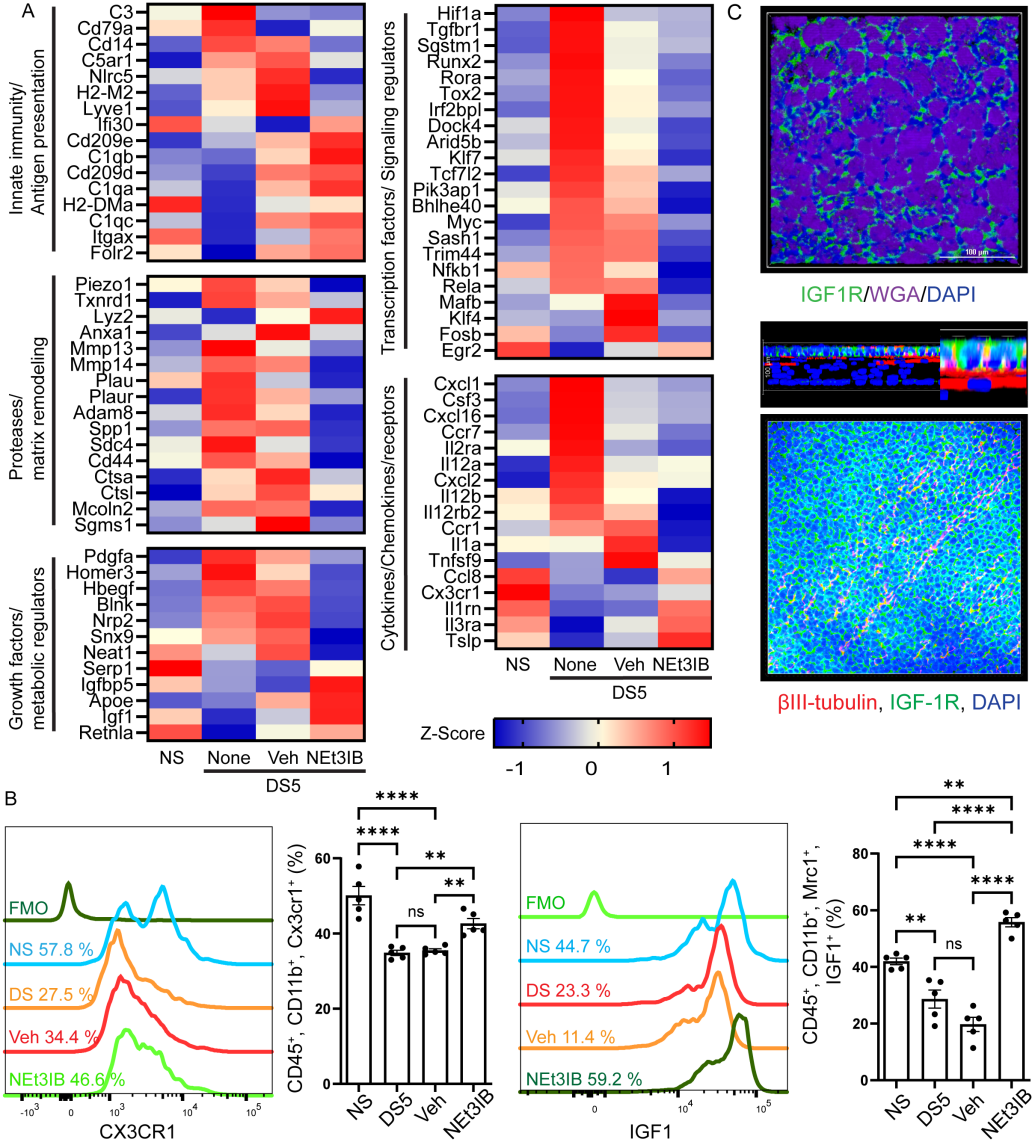
Effects of rexinoid treatment on macrophage gene and protein expression in the desiccation stress dry eye model and potential macrophage-derived IGF-1/IGF-1R signaling axis. (**A**) Heatmaps showing *z*-score–scaled expression of selected latent-time–associated genes in four treatment groups (NS, None [untreated], Veh, and NEt-3IB) in conjunctival monocyte/macrophage lineage cells sorted from CD45⁺ cells after 5 days of desiccating stress (DS5) (*P_adj_* < 0.001). Genes are organized into five functional categories. The *color scale* represents *z*-scores of normalized expression (*blue*, low; *red*, high). These data show desiccating stress–associated shifts in macrophage gene programs and their modulation by NEt-3IB, including enrichment of reparative and growth factor–related genes such as *Igf1*. Full gene names for the abbreviations are provided in Supplementary Table S3. (**B**) Representative flow cytometry histograms and quantification validating selected macrophage-associated markers in conjunctival immune cells. (*Left*) Representative histograms of CX3CR1 staining with fluorescence-minus-one (FMO) control, with corresponding quantification of the percentage of CD45⁺CD11b⁺CX3CR1⁺ cells. (*Right*) Representative histograms of IGF-1 staining with FMO control, with corresponding quantification of the percentage of CD45⁺CD11b⁺Mrc1⁺IGF-1⁺ cells. Compared with DS5 no treatment and DS5+vehicle controls, DS5+NEt-3IB increased the proportion ofx CX3CR1⁺ and Mrc1⁺IGF-1⁺ myeloid cells. Each *dot* represents one biological replicate; *bars* show mean ± SEM. Statistical significance is indicated as shown: **P < 0.01; ****P < 0.001; ****P < 0.0001; ns, not significant. (**C**) Representative immunofluorescence images showing IGF-1R/WGA/DAPI staining of wholemount conjunctiva showing surface view (*top*) and βIII-tubulin/IGF-1R/DAPI staining in the cornea (*bottom*). IGF-1R localization is shown because IGF-1 is produced by ocular surface resident macrophages, suggesting a potential macrophage-derived IGF-1/IGF-1R signaling axis acting on ocular surface epithelial and neural compartments during DS. *Scale bar*: 100 µm.

Latent time increased with desiccating stress, both without and with vehicle treatment (Supplementary Fig. S1B). The changes in gene expression observed after desiccating stress exposure in these groups are consistent with our previous findings: Inflammatory gene expression increased, and homeostatic gene expression decreased in desiccating stress.^6^ In contrast, latent time remained closer to non-stressed in the NEt-3IB–treated group. Supplementary Figure S1B shows the latent time in bar graphs with superimposed line graphs plotting expression of inflammatory genes *Il1a*, *Cxcl1*, and *Nfkb1* that increased with desiccating stress with or without vehicle and decreased with NEt-3IB treatment. The opposite pattern is seen in expression of the homeostatic/anti-inflammatory genes *Igf1*, *Il1rn*, and *Egr2*, which decreased with desiccating stress and increased with NEt-3IB treatment (Supplementary Fig. S1B). This indicates that NEt-3IB maintains the macrophage cell trajectory closer to the baseline homeostatic state. This pattern can be seen in the heatmaps of 83 DEGs (*P*_adj_ < 0.001) in the monocyte/macrophage lineage cells (Fig. 1A) that are classified into five functional groups (innate immunity/antigen presentation, protease/remodeling, growth factors/metabolic regulators, transcription factors/signaling regulators, and cytokines/chemokines/receptors). NEt-3IB increased expression of resident macrophage–associated genes (*C1qa-c*, *Folr2*, *Igf1*, *Egr2*, and *Cx3Cr1*) and decreased expression of inflammatory genes (*Cxcl1*, *Ccr7*, *Il12a,b*, and *Il1a*).

Flow cytometry (Fig. 1B) was performed on conjunctival immune cells from each experimental group (NS, DS5, DS5+vehicle, DS5+NEt-3IB) to compare the percentage of cells positive for two resident macrophage–associated proteins, CX3CR1 (fractalkine receptor) and IGF-1, whose gene expression significantly increased with NEt-3IB (Fig. 1A). High CX3CR1 protein expression is characteristic of resident macrophages,^17^ and resident macrophages are the major producers of IGF-1 cytokine in the cornea and conjunctiva.^6^^,^^18^ IGF-1 is recognized to promote epithelial health and maintain barrier function in the corneal epithelium and gut mucosa.^19^^,^^20^ IGF-1 concentration in tears has also been reported to decline with age.^21^ The percentage of both CX3CR1^+^ and IGF-1^+^ cells significantly decreased in the DS no treatment and DS5+vehicle treated groups. The percentage of CX3CR1^+^ cells returned to baseline with NEt-3IB treatment, whereas the percentage of IGF-1^+^ cells was higher than baseline in the DS5+NEt-3IB–treated group. The same pattern was seen for mean fluorescence intensity (MFI) of both proteins.

### Effects of NEt-3IB on Cornea and Conjunctival Epithelial Disease

We previously found that depletion of resident macrophages worsened ocular surface inflammation and epithelial disease in experimental dry eye.^6^ Based on the finding that NEt-3IB increases expression of resident macrophage–associated genes and proteins and suppresses inflammatory genes, we evaluated the effects of NEt-3IB on corneal and conjunctival epithelial disease in the desiccating stress model.

**Figure 2.**
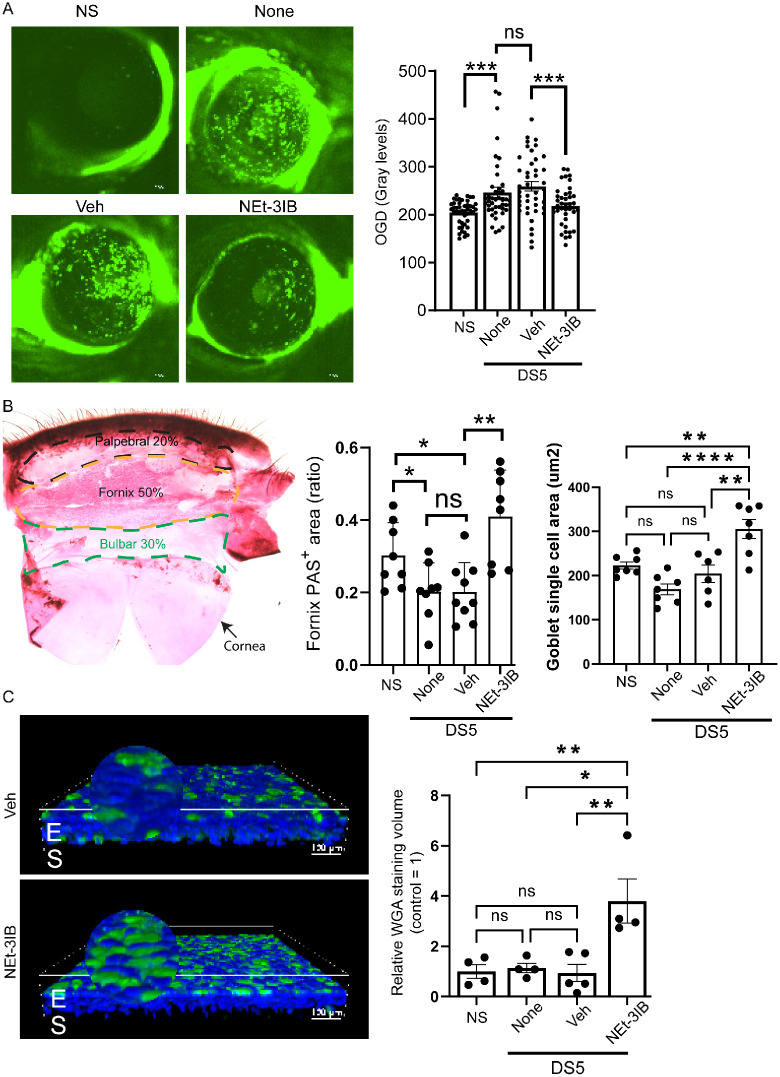
Effects of desiccation stress and NEt-3IB treatment on corneal epithelial permeability and goblet cell density in mice. (**A**) Representative Oregon Green Dextran (OGD) fluorescence images of corneas from mice subjected to 5 days of desiccation stress (DS5) and treated three times daily with vehicle (Veh) or NEt-3IB, or no treatment (none) compared to non-stressed (NS) controls. Increased fluorescence indicates higher corneal epithelial permeability. Data are presented as mean ± SEM (*n* = 19 biological replicates, or 38 eyes). (**B**) (*Left*) Representative PAS-stained conjunctival wholemount showing goblet cells (*magenta*) in palpebral (*black dashed line*), fornix (*red dashed line*), and bulbar (*green dashed line*) regions. (*Center*) PAS^+^ area in the fornix region expressed as a ratio of the PAS^+^ area/total area in all groups was measured as described in the Materials and Methods. (*Right*) Quantification of goblet cell single-cell area (µm^2^) in all groups. Data are shown as mean ± SEM (*n* = 7 to 9 biological replicates). (**C**) (*Left*) 3D confocal images of WGA-stained goblet cells in WGA-stained conjunctival wholemounts in vehicle and NEt-3IB treated groups. (*Right*) The 3D volume of WGA^+^ goblet cell glycoprotein in WGA lectin-stained conjunctival wholemounts in the four treatment groups was measured as described in the Materials and Methods. Volumes are presented as the relative ratio (fold change) versus the control group. E, epithelium; S, stroma. Statistical significance was determined by one-way ANOVA with post hoc analysis (*n* = 4 or 5 biological replicates). *P < 0.05; **P < 0.01; ***P < 0.001; ns, not significant.

Compared to the non-stressed group, we found that corneal permeability to the 70-kDa fluorescent tracer molecule, Oregon Green Dextran (OGD), significantly increased in the DS5 (none) group. Oregon Green Dextran staining in the vehicle group was not different than in the DS5 group, but it was significantly higher than the NEt-3IB group. This indicates that rexinoid treatment preserves corneal barrier function in response to desiccating stress (Fig. 2A). We also evaluated if NEt-3IB had any adverse effects on the intraepithelial nerve plexus using Sholl analysis. Like our previous finding, there was no reduction in nerve density in the DS5 group nor in the NEt-3IB group compared to non-stressed; however, there is a significant reduction in nerve density in the vehicle group (Supplementary Fig. S3). This indicates that NEt-3IB is not toxic to the nerve plexus.

The effects of NEt-3IB treatment on conjunctival goblet cell area, cell number, and cell volume were evaluated. Shi et al.^16^ reported that, in wholemounts of the entire conjunctival surface, goblet cell density was highest in the conjunctival forniceal region. They also found that topical retinoic acid treatment significantly increased goblet cell number. We used this same wholemount method and found that the total goblet cell area (percentage of PAS^+^ cells/entire area) was significantly lower in the no treatment and vehicle desiccating stress groups and significantly higher in the NEt-3IB group compared to vehicle (Fig. 2B). The same pattern was also seen for mean individual cell area. We also evaluated 3D volume of WGA-stained glycoproteins in conjunctival wholemounts. The NEt-3IB group had significantly greater mean volume of WGA-stained glycoprotein compared to the other three groups (Fig. 2C).

Because RXR⍺ is expressed in corneal and conjunctival epithelium,^22^^,^^23^ we compared the effects of conditional RXR⍺ knockout in both corneal epithelium cells (K12rtTA;tetO-Cre,Rxra^flox/flox^) and monocyte/macrophage lineage cells (Lyz2Rxra^flox/flox^). Corneal epithelial knockout had no effect on corneal epithelial barrier function compared to the recombinase negative control without or with doxycycline induction (Supplementary Fig. S4A). There was also no difference in density of the corneal intraepithelial nerve plexus among wild-type C57BL/6 (B6), control, and conditional knockout using Sholl analysis (Supplementary Fig. S4B). On the other hand, conditional knockout in macrophage cells worsened barrier function (increased fluorescent lectin uptake) (Supplementary Fig. S4A). Additionally, rexinoid treatment of cultured conjunctival epithelium had no effect on MUC5AC immunoreactivity or gene expression (Supplementary Fig. S5).

### Effects of Monocyte Adoptive Transfer on Goblet Cell Density

**Figure 3.**
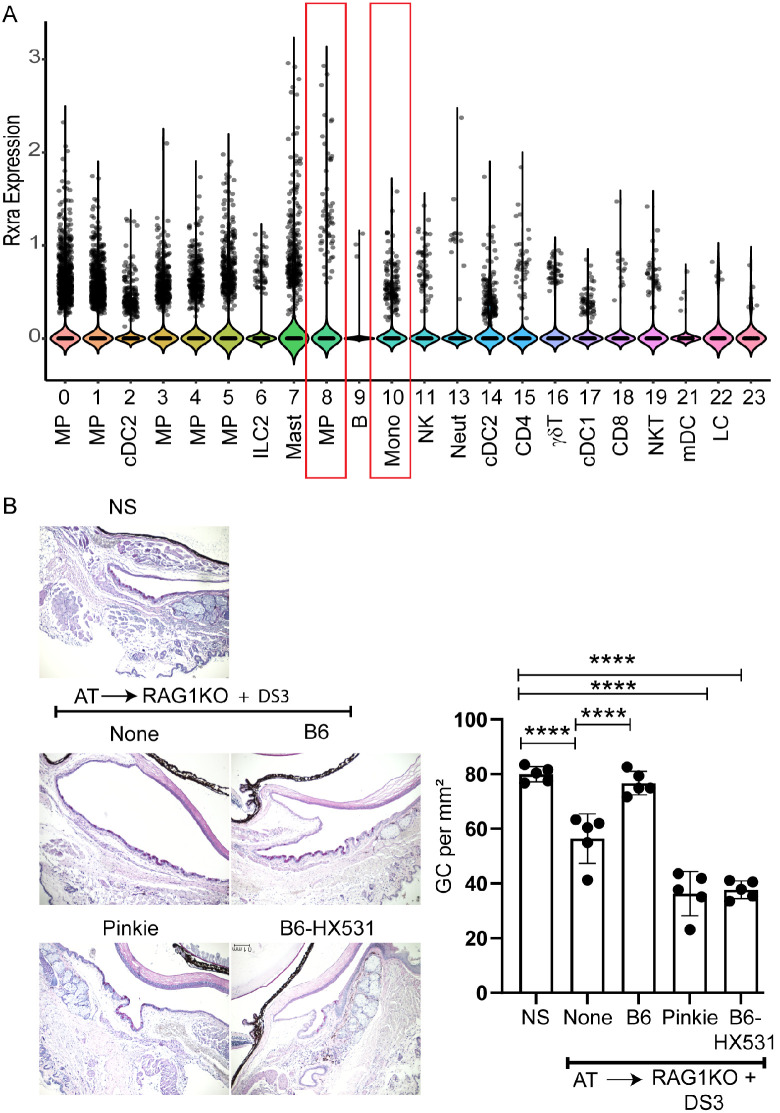
Adoptive transfer of monocytes modulates conjunctival goblet cell density under desiccating stress. (**A**) Violin plot of RXRα expression in conjunctival immune cells, macrophages (MP), and monocytes (mono) in *boxes*. cDC2, conventional dendritic cell 2; ILC2, innate lymphoid cell 2; Mast, mast cell; B, B cells; NK, natural killer cell; Neut, neutrophil; cDC2, conventional dendritic cell 2; T cells, CD4, CD8, ⍺βTCR, γδTCR, and NKT; cDC1, conventional DC1; mDC, myeloid dendritic cells; LC, Langerhan cell. (**B**) Monocytes were purified from bone marrow of C57BL/6 (B6) mice, B6 mice treated with the RXRα antagonist HX531, or Pinkie strain carrying an RXRα mutation. Approximately 2 × 10^6^ purified monocytes were injected into the subconjunctival space of RAG1KO recipient mice. As controls, RAG1KO mice received no cell transfer (none) or were left untreated (NS). Following adoptive transfer (AT), mice were subjected to 3 days of desiccating stress (DS3) before eyes were collected and conjunctival tissue analyzed. Representative PAS–stained sections of conjunctiva are shown for each treatment group (*left*). Quantification of conjunctival goblet cell (GC) density (cells per mm^2^) is presented on the *right* (mean ± SEM, *n* = 5 or 6 per group). Adoptive transfer of B6 monocytes partially restored goblet cell density compared to the None group, whereas monocytes from Pinkie or HX531-treated B6 failed to confer protection. Statistical significance was determined by one-way ANOVA with post hoc analysis. ****P < 0.0001.

To evaluate if monocytes can suppress desiccation-induced conjunctival goblet cell loss, we performed adoptive transfer of purified bone marrow monocytes from C57BL/6 donors with and without treatment with RXR⍺ agonist HX531 and from the RXR⍺ mutant Pinkie strain to B- and T-cell–deficient RAG1KO recipients, then subjected them to desiccating stress. As shown in the violin plot (Fig. 3A), monocytes and macrophages expressed RXR⍺ as we previously reported.^7^^,^^24^ We found that goblet cell density was significantly higher than desiccating stress alone in eyes receiving WT B6 cells, and it was significantly lower in the Pinkie and B6 HX531 treated recipients (Fig. 3B).

### Comparison of Dexamethasone and NEt-3IB on LPS-Stimulated Monocytes

**Figure 4.**
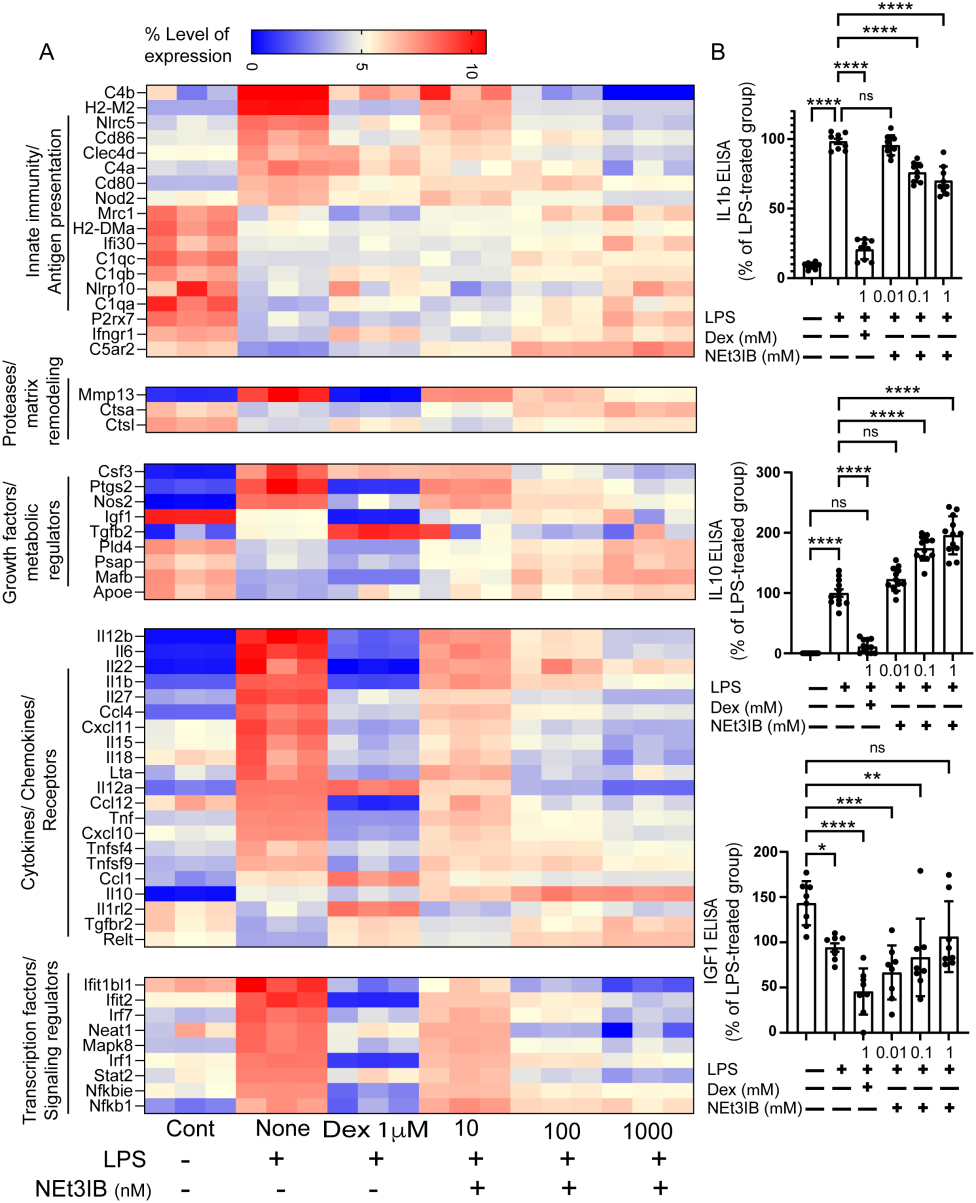
Gene expression profiles of monocytes treated with dexamethasone or NEt-3IB. (**A**) Purified mouse bone marrow–derived monocytes were cultured under different treatment conditions and analyzed by bulk RNA sequencing. Cells were left untreated (Cont), stimulated with LPS alone (None), or treated with LPS in combination with either dexamethasone (Dex, 1 µM) or NEt-3IB at increasing concentrations (10, 100, 1000 nM). Heatmap shows normalized expression levels of selected genes grouped into five functional categories (*left*). Expression values are scaled by row, with *red* indicating higher expression and *blue* indicating lower expression relative to the mean (scale, 0–10). Dexamethasone treatment broadly suppressed pro-inflammatory cytokines/chemokines, whereas NEt-3IB exerted a dose-dependent modulatory effect on both innate immune genes and transcriptional regulators (*n* = 3 biological replicates per group). (**B**) ELISA quantification of IL-1β, IL-10, and IGF-1 production by purified monocytes after pretreatment with Dex (1 µM) or NEt-3IB (0.01, 0.1, or 1 µM) followed by LPS stimulation (0.5 µg/mL, 20 hours). Cytokine levels are presented as a percentage of the LPS-treated group. NEt-3IB significantly reduced IL-1β production and increased IL-10 and IGF-1 levels in a dose-dependent manner. Each *dot* represents one biological replicate; *bars* show mean ± SEM. Statistical significance was determined by one-way ANOVA with post hoc analysis. ****P < 0.0001.

Because corticosteroids are an approved and often prescribed therapy for dry eye,^25^^,^^26^ we compared the effects of the aqueous soluble corticosteroid dexamethasone (1 µM) with NEt-3IB (0.01, 0.1, and 1µM) on gene expression in LPS-stimulated bone marrow–derived monocytes by RNA sequencing. The DEGs were classified in the same five functional groups/pathways used for gene annotation in conjunctival cells (Fig. 1C). We found a dose-dependent decrease in inflammatory gene expression with NEt-3IB treatment. The magnitude of suppression with 1-µM NEt-3IB was similar, but slightly lower than that observed with 1-µM dexamethasone (Fig. 4A). Increased expression of homeostatic and resident macrophage–associated genes (*Mrc1*, *Igf1*, and *Il10*) was also noted with NEt-3IB, but this was not observed with dexamethasone. We also noted a dose-dependent decrease in IL-1β and increase in IL-10 and IGF-1 proteins in supernatants of NEt-3IB–treated monocyte cultures (Fig. 4B).

Dexamethasone is recognized to increase intraocular pressure in steroid responders, which limits its chronic use for treating ocular surface inflammation in dry eye. We compared the effects on intraocular pressure measured at each time point in mice treated topically with either dexamethasone or NEt-3IB (both 1 µM), four times daily for 20 days. Compared to baseline, intraocular pressure was significantly increased (*P* < 0.0001) at all time points in the dexamethasone group and at days 3 and 10 in the NEt-3IB group (*P* < 0.0001). Intraocular pressure in the dexamethasone group was significantly higher than in the NEt-3IB group after 1 day, 10 days, and 20 days of treatment (Supplementary Fig. S6).

## Discussion

Tissue-resident macrophages are recognized to maintain homeostasis, defend against pathogens, suppress inflammation, and repair tissue damage.^27^ Macrophage phagocytosis and efferocytosis (clearance of apoptotic cells) suppresses inflammation and triggers production of anti-inflammatory factors such as IL-10.^3^ RXR⍺ and partner receptors (i.e., PPARγ) regulate the expression of genes involved in these resident macrophage functions, and we found that these are among the most active pathways in conjunctival resident macrophages.^6^

This study evaluated effects of rexinoid NEt-3IB, a water-soluble sodium salt, on conjunctival immune cell gene expression and severity of ocular surface disease. We found that, in eyes subjected to short-term desiccating stress for 5 days, NEt-3IB maintained a resident macrophage gene expression pattern by suppressing expression of inflammatory mediators and increasing expression of homeostatic/regenerative factors (*Egr2*, *Folr2*, *Cx3cr1*, and *Igf1*). In support of the latent time findings, an increased percentage (and MFI) of conjunctival cells expressing the resident macrophage markers CX3CR1 and IGF-1 was noted by flow cytometry in the NEt-3IB group. NEt-3IB also maintained corneal barrier function and conjunctival goblet cell size, area, and density. We previously found that *Igf1* is highly expressed by resident macrophages in the cornea and conjunctiva, and IGF-1 receptors are expressed by the conjunctival and corneal epithelia.^6^^,^^10^ Together with substance P, IGF-1 has been reported to restore corneal epithelial barrier function in a rat model of neurotrophic keratitis.^20^ These findings suggest that rexinoid conditioning of resident macrophages, possibly through an IGF-1/IGF-1R signaling axis, may be responsible for the observed improvement in ocular surface disease, but additional experiments are needed to demonstrate causality. The effects of NEt-3IB were evaluated in an acute dry eye model in female C57BL/6 mice because we and others have found that the desiccating stress model does not cause dry eye disease in male mice.^28^ Dry eye disease is often chronic, and additional studies are needed to determine if this rexinoid is effective with chronic administration or if it is effective in treating pre-existing dry eye.

Additionally, NEt-3IB dose dependently reduced expression of dry eye–relevant macrophages and inflammatory mediators (*Il1b*, *Il6*, *Il12a,b*, *Cxcl10*, and *Il18*) produced by stimulated bone marrow–derived monocyte cultures such as the corticosteroid dexamethasone. However, in contrast to cells treated with the dexamethasone at the same concentration of 1 µM, NEt-3IB also increased expression of resident macrophage markers (*Mrc1*, *Apoe*, *Igf1*, and *Il10*) in LPS-stimulated cells. Only a single concentration of dexamethasone was used for comparison, and it is possible that other concentrations may have a different effect on expression of resident macrophage–associated genes. This concentration of dexamethasone significantly increased intraocular pressure at three of four time points over 20 days of treatment, whereas NEt-3IB increased pressure at only one time point. Determining whether the risk of intraocular pressure elevation is lower with rexinoid compared to corticosteroid requires additional studies to evaluate a range of concentrations and of longer duration.

Most rexinoids (including 9-*cis*-retinoic acid) are molecules with a hydrophobic moiety characterized by a 1,1,4,4-tetramethyltetralin group and an acidic moiety as the pharmacophore which renders them highly lipophilic and poorly soluble in aqueous solutions.^29^ In NEt-3IB, the rexinoid used in this study, the 1,1,4,4-tetramethyltetralin group was converted to an aromatic ring with a polar alkoxy group that rendered it more soluble in aqueous solutions than existing rexinoids while maintaining sufficient rexinoid activity.^30^^,^^31^ To further enhance water solubility, NEt-3IB was converted to a sodium salt (Supplementary Methods). Its aqueous solubility (5 mM) makes it a better candidate than earlier molecules for topical ocular administration in an aqueous solution. NEt-3IB has a higher binding affinity toward RXRα (*K_d_* = 9 nM) than bexarotene (*K_d_* = 144 nM; note that lower means higher affinity), which is a U.S. Food and Drug Administration–approved systemic rexinoid for treatment of cutaneous T-cell lymphoma (CTCL).^31^ Furthermore, NEt-3IB has much lower retinoid acid receptor (RAR) activity than bexarotene. In preclinical studies, NEt-3IB was found to significantly improve disease in a mouse colitis model.^29^ In the current study, we did not observe corneal epithelial toxicity from topically applied NEt-3IB measured by fluorescent dextran permeability or epithelial nerve plexus morphology by immunostaining. Of note, retinol, which is a precursor for retinoic acid that binds to the RAR and can serve as a heterodimeric partner with RXR, has also been reported to increase goblet cell density.^16^^,^^32^^,^^33^ Based on our preliminary experiment in mice, NEt-3IB caused a slight but significant intraocular pressure increase at days 3 and 10, but this was significantly less than the increase induced by the corticosteroid dexamethasone, which was used at a lower concentration than used therapeutically in humans and previous mouse models.^34^

**Figure 5.**
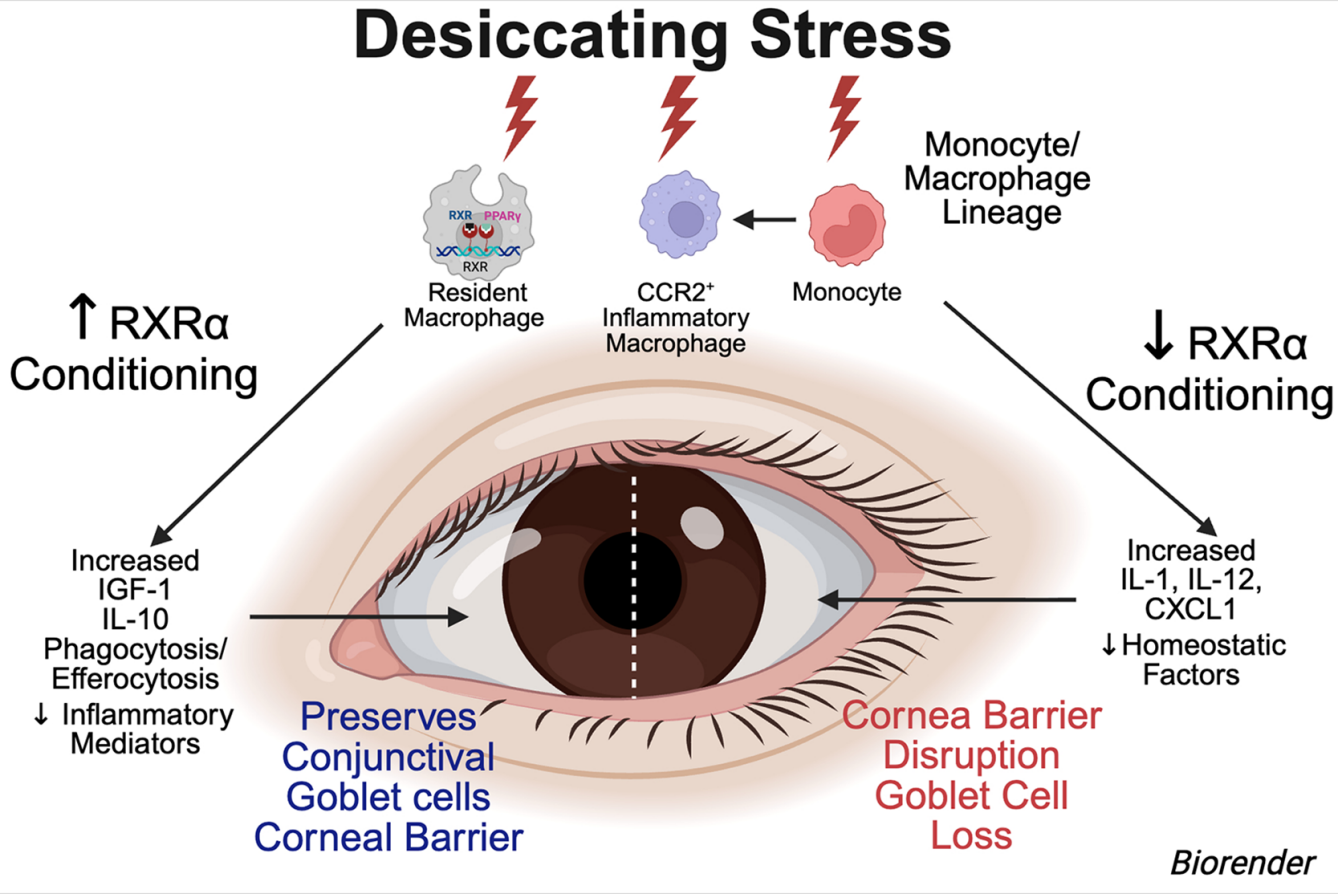
The proposed mechanism of action responsible for therapeutic activity is rexinoid conditioning of resident macrophage, monocytes recruited from the blood, and monocyte-derived inflammatory macrophages.

We previously reported that depletion of resident macrophages with mannosylated clodronate worsened the severity of desiccating stress–induced inflammation and ocular surface disease.^6^ In the current study, we found that topically applied NEt-3IB suppressed expression of inflammatory genes and stimulated expression of reparative genes associated with resident macrophage, as well as maintaining corneal barrier and conjunctival goblet cell area during exposure to desiccating stress. The proposed mechanism of action responsible for rexinoid therapeutic activity is conditioning resident or recruited macrophages to express higher levels of homeostatic/regenerative factors and lower levels of inflammatory mediators (Fig. 5).

We evaluated the effects of NEt-3IB on gene expression at only one time point during exposure to desiccating stress. Macrophage differentiation is plastic and influenced by environmental factors. Using single-cell sequencing we previously reported changes in macrophage gene expression over 1 to 10 days of desiccating stress with increased expression of inflammatory genes in CCR2^+^ macrophages and increases in homeostatic and phagocytosis genes in the CCR2^–^ resident macrophages.^6^ Based on this plasticity, the effects of NEt-3IB on gene expression may be different if evaluated at other time points during desiccating stress.

Supporting the concept that regenerative and anti-inflammatory properties of conjunctival resident macrophages can suppress severity of desiccation-induced goblet cell loss, we found that adoptively transferred bone marrow–derived monocytes from wild-type B6 maintained goblet cell number during desiccating stress; however, monocytes from the RXR⍺ Pinkie mutant or from the B6 strain treated with the RXR⍺ antagonist HX531 had a significantly lower number of goblet cells. These data suggest that monocytes or monocyte-derived macrophages can counter the deleterious effects of dry eye on conjunctival goblet cells.

## Supplementary Material

Supplement 1

Supplement 2

Supplement 3

Supplement 4

Supplement 5
