# Supplementary material for: Rexinoid NEt-3IB Promotes Resident Macrophage Gene Expression and Mitigates Desiccation-Induced Ocular Surface Disease
Source: Invest Ophthalmol Vis Sci. 2026 Apr 14;67(4):31. doi: 10.1167/iovs.67.4.31 (PMC13089652; doi:10.1167/iovs.67.4.31)
Supplement: Supplement 1 [file iovs-67-4-31_s001.docx]

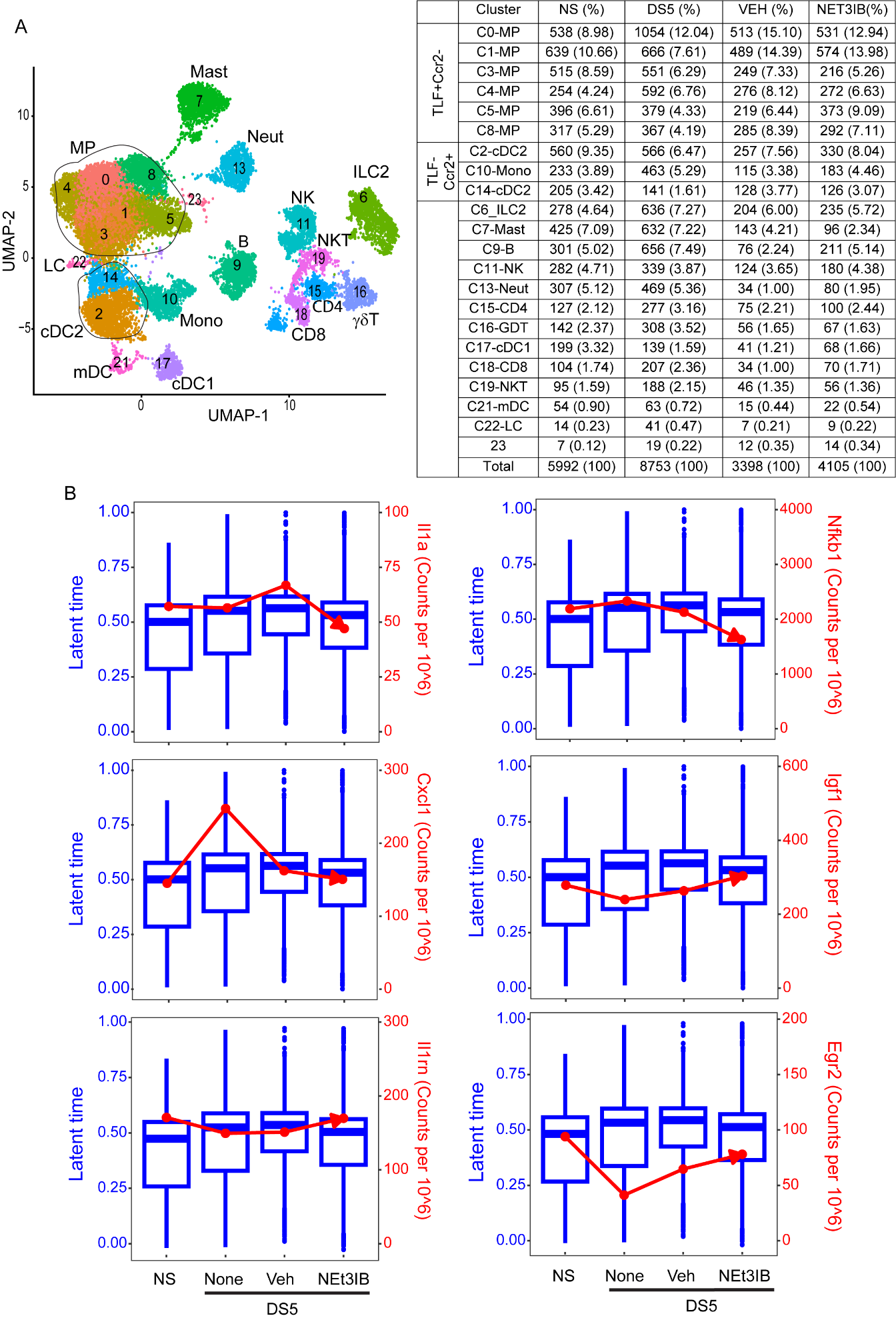


**Figure S1. Conjunctival immune cell clustering and macrophage latent-time analysis in the desiccation stress dry eye model.**

A. UMAP visualization of single-cell RNA-seq data showing major immune cell populations defined below. Clusters 0,1,3,4,5,8 (upper outline) are TLR^+^CCR2^-^ cells, clusters 2 and 14 (lower outline) are TLR^-^CCR2^+^ cells, The table on the right illustrates the number and relative proportion of each cluster across experimental conditions (NS=non-stressed; DS5 = desiccating stress for 5 days (DS5) without treatment (none); Veh= DS5 + vehicle 3 times per day; NEt-3IB = DS5 + NEt-3IB 5 μM 3 times per day). Cells were obtained from 8 mice per group.

B. Latent time analysis of selected inflammatory (*Il1a*, *Nfkb1*, *Cxcl1)* and regulatory genes (*Igf1*, *Il1rn*, and *Egr2*) in macrophages. Blue boxplots indicate the distribution of latent time scores, reflecting pseudotemporal ordering of transcriptional programs. Red lines represent normalized gene expression counts (per 10⁶) across latent time. These data illustrate dynamic changes in inflammatory and homeostatic/reparative macrophage-associated programs along the latent-time trajectory in the conjunctiva during desiccation stress and show modulation by NEt3IB, including increased Igf1 and Egr2 expression.

MP = macrophage, Mono = monocyte, cDC2 = monocyte derived dendritic cell, Neut = neutrophil, Mast = mast cell, B = B cell, T cells = CD4, CD8, ⍺βTCR, γδTCR, NKT, NK = natural killer cell, ILC2 = innate lymphoid cell, cDC1 = = conventional DC1, mDC = myeloid dendritic cell. Numbers denote individual clusters.


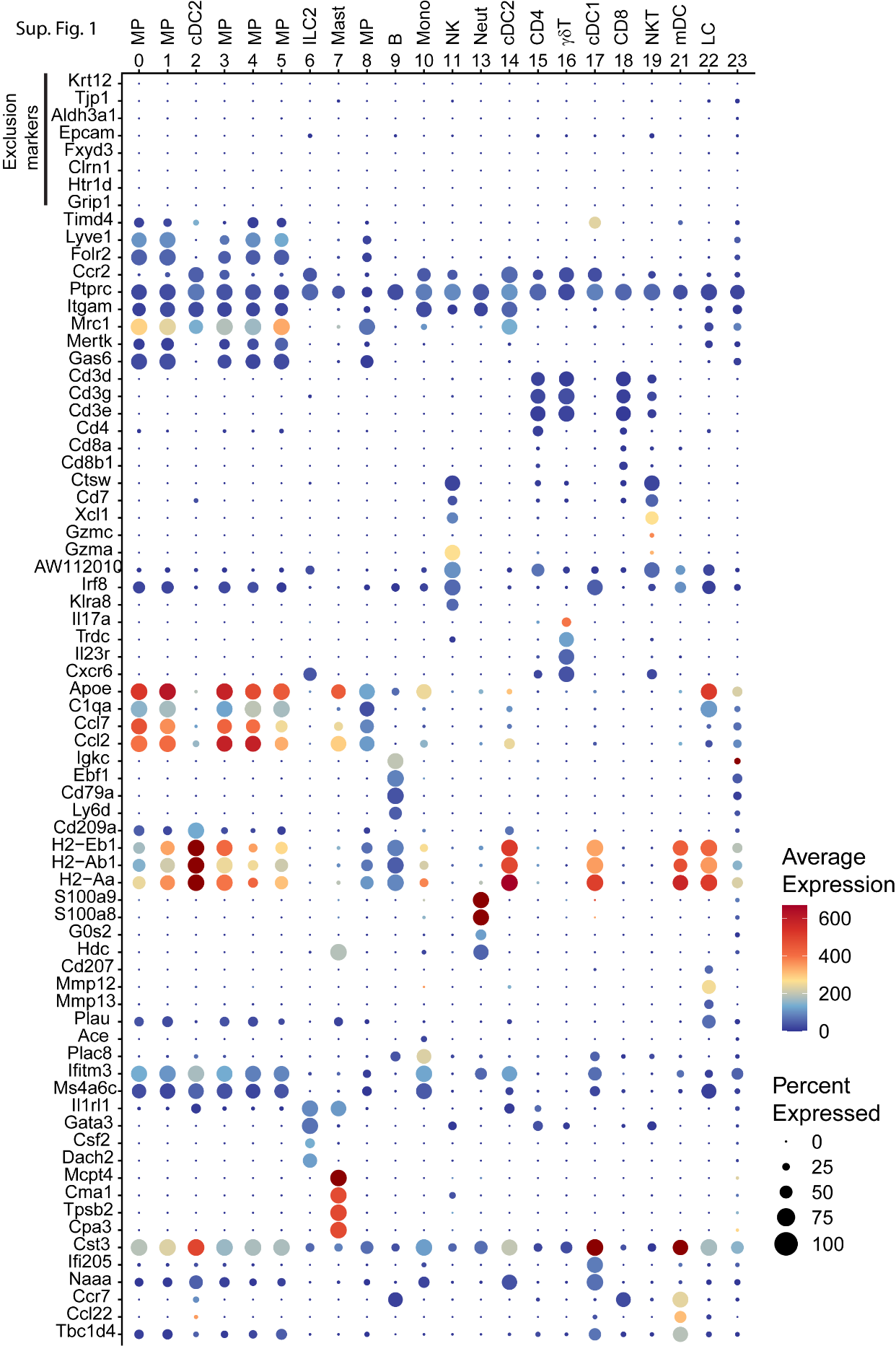


**Figure S2.** Dot plot showing relative levels of expression of *Timd4, Lyve-1, Folr2* (TLF) and *Ccr2* as well as inflammatory, homeostatic and phagocytic genes in conjunctival immune cells.


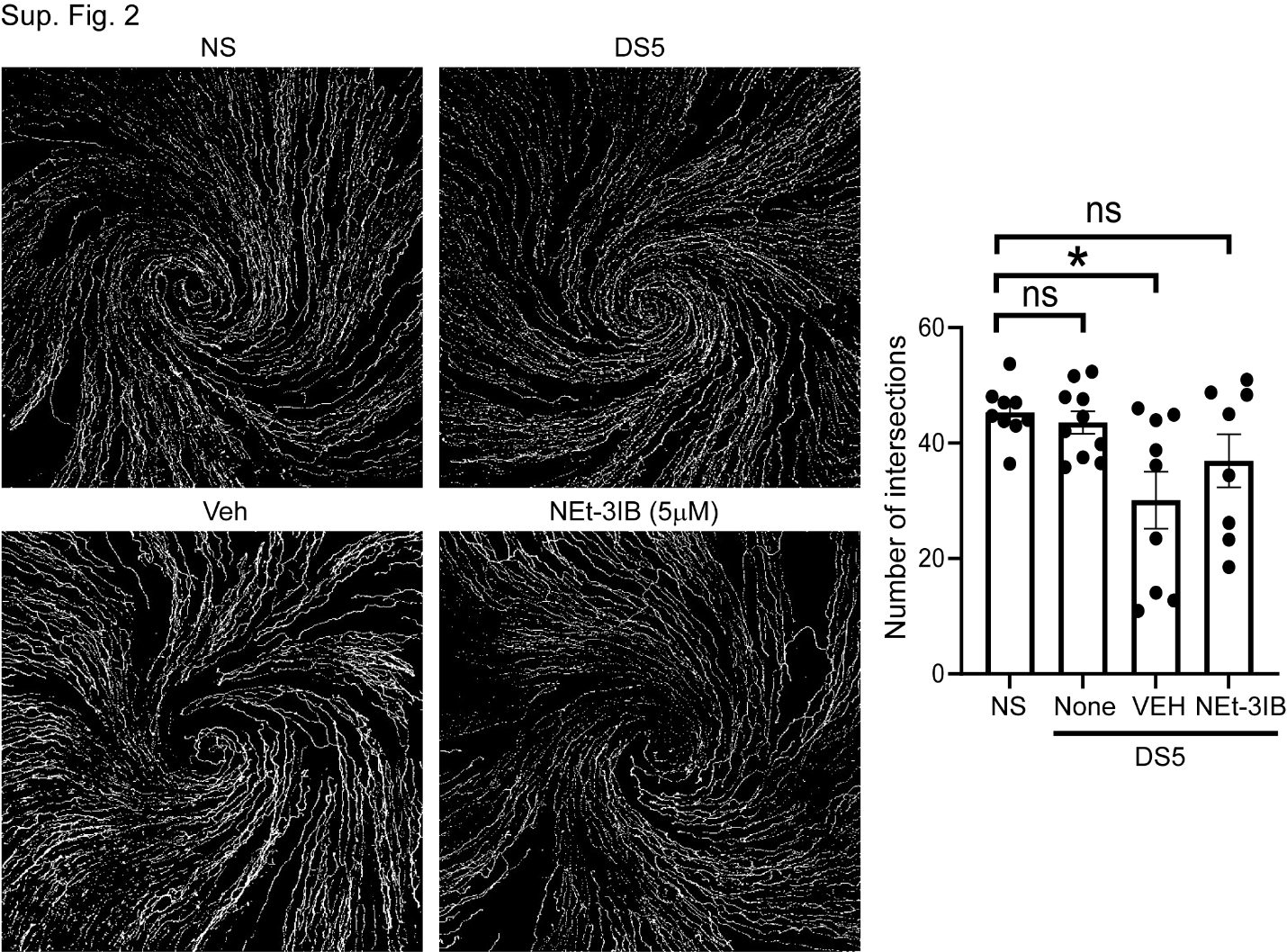


**Figure S3.** **Representative corneal nerve tracings and Sholl analysis**. Whole-mount corneas from NS controls, DS5-treated mice without eye drops (None), and DS5 mice treated with vehicle or NEt-3IB (5 µM) were immunostained and processed for nerve tracing. Sholl analysis was performed to quantify corneal nerve density by measuring the number of neurite intersections at concentric radii centered on the vortex region. Quantification (right) shows the number of intersections per cornea. Each dot represents one animal; bars indicate mean ± SEM. Statistical comparisons were performed using one-way ANOVA with post hoc testing. n=9 biological replicates.


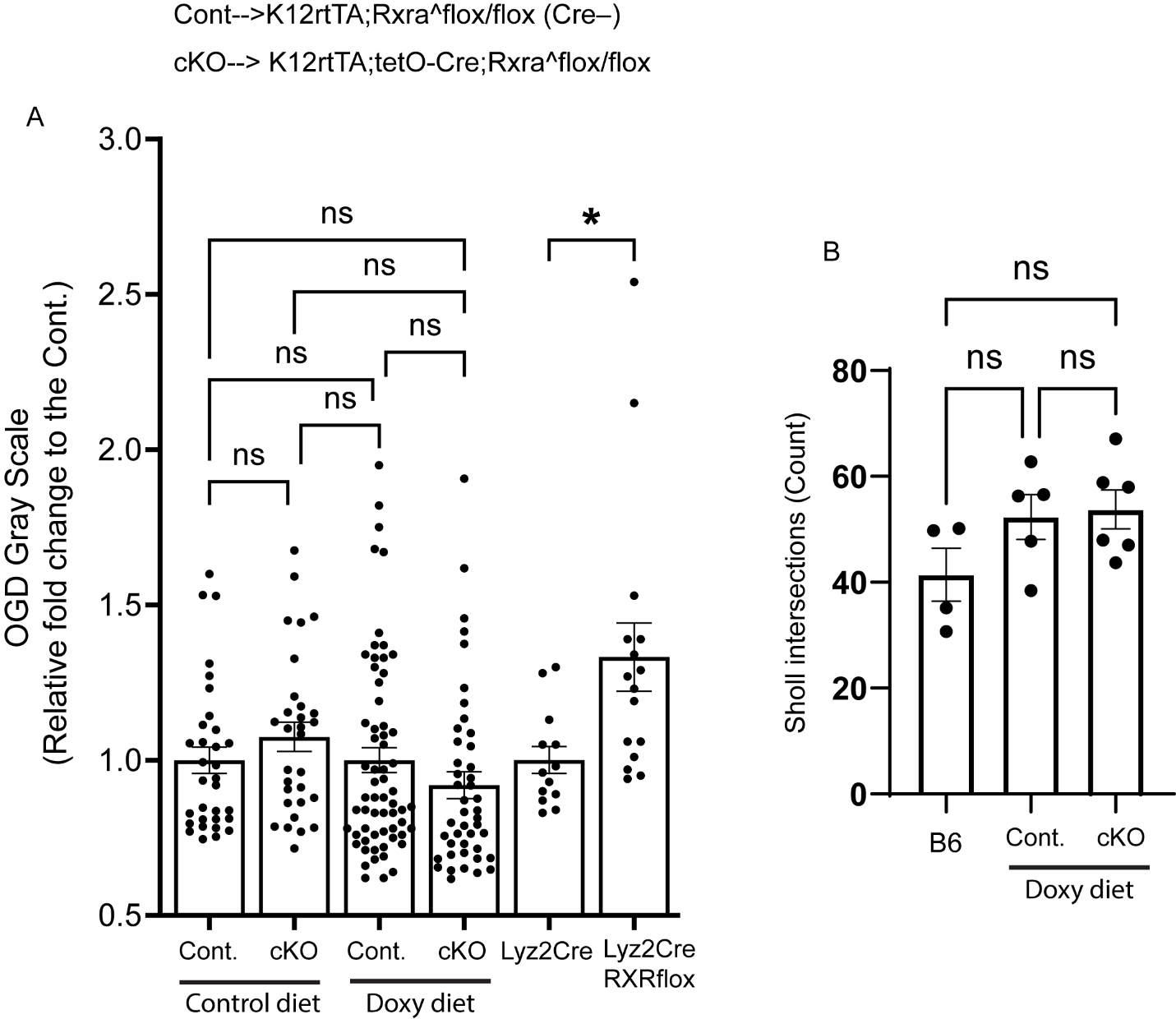


**Figure S4.** Effects of conditional RXR⍺ gene knockout in the corneal epithelium and macrophages on corneal barrier function and nerve morphology.

A. Corneal epithelial permeability to the fluorescent molecule 70kDa Oregon Green Dextran (OGD) as a measure of barrier function was evaluated in a strain with a doxycycline inducible conditional RXR⍺ gene knockout (KO) in the corneal epithelium. Recombinase negative (Cre^-^) control (K12rtTA;tetO-Cre,Rxra˄flox/flox) and Cre^+^ K12rtTA;tetO-Cre,Rxra˄flox/flox mice with and without doxycycline induction and in a strain with conditional KO in monocyte/macrophage lineage cells (Lyz2CreRXRflox/flox). Cre in this strain is constitutively expressed There was no difference in corneal barrier function (relative fold change in grey scale fluorescence as described in the methods) with epithelial RXR⍺ deletion; however, RXR⍺ deletion in the macrophage lineage cells disrupted the barrier and increased fluorescent tracer uptake in the corneal epithelium. Statistical comparisons were performed using one-way ANOVA with post hoc testing; bars indicate mean ± SEM; n= 30-63 for the epithelial conditional KO and n= 13-15 for the macrophage conditional KO. (*p < 0.05)

B. Sholl analysis of corneal nerves in 6-8 week old control C57BL/6 (B6) and doxycycline induced Cre- (Cont) and Cre+ cKO K12rtTA;tetO-Cre,Rxra˄flox/flox strain. Corneas were immunostained and processed for nerve tracing. Sholl analysis was performed to quantify corneal nerve density by measuring the number of neurite intersections at concentric radii centered on the vortex region. Graph shows the number of intersections per cornea on the Y axis. Each dot represents one animal; bars indicate mean ± SEM. Statistical comparisons were performed using one-way ANOVA with post hoc testing. n= 4-6 biological replicates.


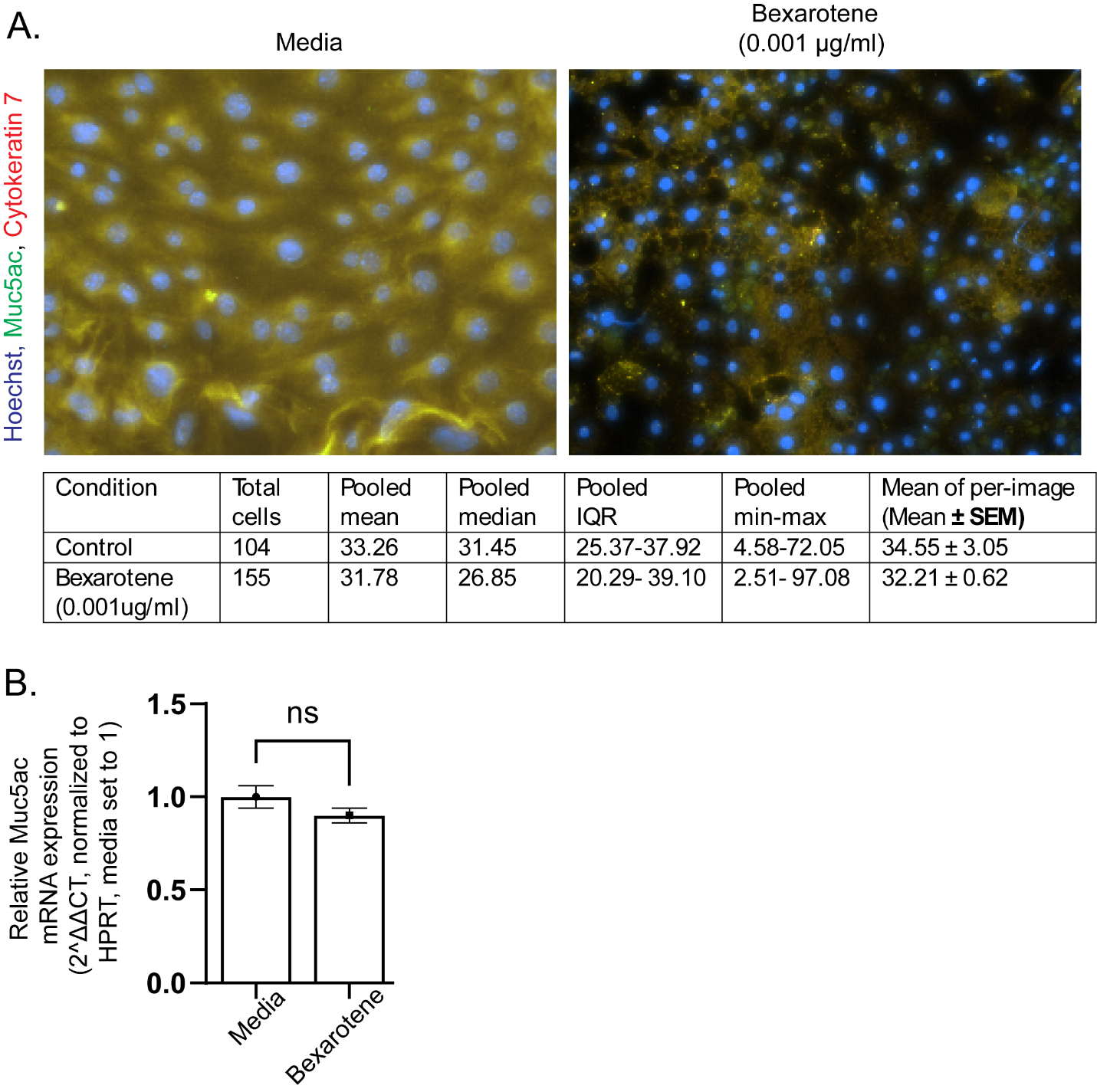


Figure S5. Effects of Rexinoid Bexarotene on mucin production by cultured conjunctival epithelium.

A.Conjunctival epithelial explants were cultured with or without the rexinoid bexarotene. MUC5AC immunofluorescence was quantified using a uniform automated workflow. Nuclei were identified by DAPI staining, and per‑cell mucin intensity was measured using a standardized perinuclear ring region. Background fluorescence was subtracted uniformly across images. The same analysis parameters were applied to all samples. Bexarotene treatment did not increase per‑cell MUC5AC intensity compared with media controls, indicating no detectable upregulation of MUC5AC expression under these conditions.

B.Relative Muc5ac mRNA expression measured by quantitative real‑time PCR*.* Bar graph shows fold‑change in Muc5ac transcript levels in cells treated with bexarotene (0.001 μg/mL) compared with media‑only controls. Gene expression was quantified using the 2^ΔΔCT method, normalized to HPRT, and expressed relative to the media condition set to 1. Data are presented as mean ± SEM. Statistical significance was assessed using an unpaired test; ns indicates no significant difference between groups.


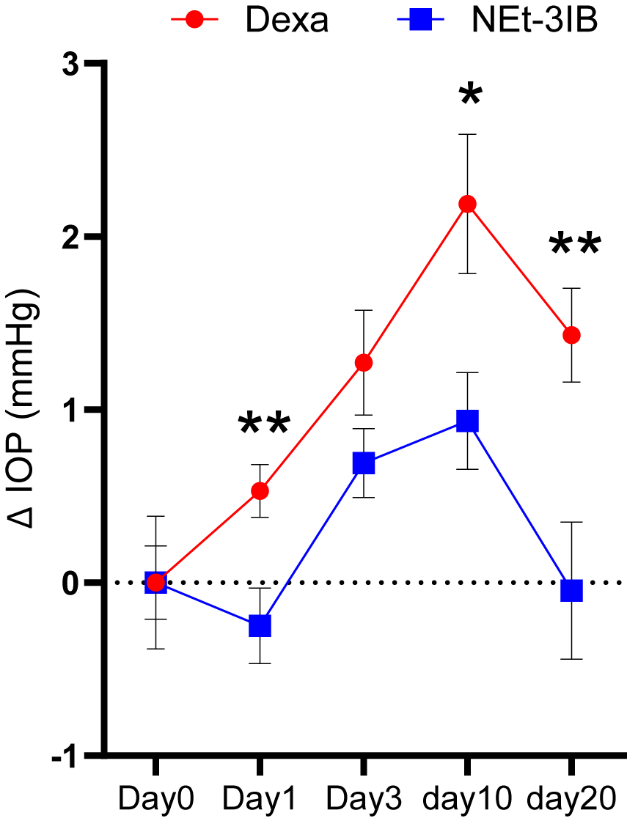


Figure S6. Comparison of NEt-3IB and dexamethasone on intraocular pressure (IOP). IOP was measured in both eyes of 8-week-old female C57BL/6 mice (n=5 per group) between 10:00AM to 12:00PM at days 0, 1, 3, 10 and 20 of topical administration of NEt-3IB 5uM or dexamethasone 1uM (Dexa) 3 times per day. Intraocular measurements were taken with an iCare IC200 tonometer (North Carolina, USA). Three pressure measurements were taken from each eye at each time point and the average each of the 6 measurements from both eyes was used for statistical comparison. IOP in the NEt-3IB was significantly higher than baseline at days 3 and 10, and Dexa was higher than baseline at all timepoints. Between group comparisons are marked. Statistical significance was determined by ANOVA with post-hoc analyses. Data are shown as mean ± SEM. * p< 0.01; ** p<0.001.

Supplementary File

Table S1. Genex that are positively and negatively correlated with latent time.

Table S2. Differentially expressed genes in non-monocyte/macrophage lineage cells.

Table S3. Full gene names for abbreviations used in Fig. 1A heatmap

Supplementary Methods. Synthesis of NEt-3IB
