# Supplementary material for: Rexinoid NEt-3IB Promotes Resident Macrophage Gene Expression and Mitigates Desiccation-Induced Ocular Surface Disease
Source: Invest Ophthalmol Vis Sci. 2026 Apr 14;67(4):31. doi: 10.1167/iovs.67.4.31 (PMC13089652; doi:10.1167/iovs.67.4.31)
Supplement: Supplement 2 [file iovs-67-4-31_s002.docx]

**Supplemental Methods**

1. **Analytical Methods**

1H-NMR (600 MHz) spectra were recorded on a Varian NMR System PS600 at room temperature. DMSO-*d*6 was used as the solvent for all routine NMR measurements. Chemical shifts are reported in ppm relative to the respective deuterated solvent peak, δ 7.26 ppm for 1H-NMR, and coupling constants are given in Hz. Powder X-ray diffraction data were collected using a RIGAKU-TTRIII-MTA diffractometer. This experiment was performed using a PerkinElmer, Inc. 2400II Elemental Analyzer. The combustion tube and reduction tube temperatures were set at 1253.15 and 923.15 K, respectively. Blank measurements were performed in the presence or absence of oxygen, and the blank signal was stabilized. Each test sample (1.5–3 mg) was accurately weighed and quantitatively converted into H2O, CO2, N2 by combustion decomposition. The contents of C, H, N, etc. were determined using a thermal conductivity detector for each component. Separately, the elemental composition of the test sample was calculated from the calibration curve for each element created using standard samples. The melting point of each sample was measured using a hot stage melting point measuring device (Yanagimoto Seisakusho, Japan). The measurement was performed three times for each sample, and the melting point range was recorded. Finely ground samples were loaded into a 0.7 mm borosilicate glass capillary and mounted on the diffractometer operating in transmission geometry, and equipped with a Johansson monochromator using Cu Kα radiation and Lynxeye detector. An Oxford Cryosystems Cryostream was used to control the temperature of the sample prior to data collection. Data were collected over the angular range 5 ≤ 2θ/θ° ≤ 60 at 2°/min.

1. **Synthesis of NEt-3IB sodium salt (NEt-3IB·Na)**

NEt-3IB was synthesized in our laboratory according to Reference 1.

NEt-3IB was dried overnight in a vacuum oven preheated to 40°C. Dry MeOH (10 mL) was placed in a 50 mL round-bottom flask equipped with a stir bar. NEt-3IB (1.00 g, 2.81 mmol) was added with stirring (250 rpm) to give a suspension. NaHCO₃ (2.81 mmol, 1.0 equiv) was then added, and the suspension was stirred at 40 °C under an argon atmosphere until a clear solution was obtained. The solvent was removed under reduced pressure, and ultrapure water (Milli-Q, 20 mL per gram of starting material) was added to give a suspension. The mixture was heated until the solution became clear and then allowed to stand at room temperature. The mixture was further kept in a refrigerator (0.5°C) to induce crystallization. The resulting solid was collected by suction filtration and dried under reduced pressure at 40 °C overnight to afford the sodium salt of NEt-3IB (NEt-3IB·Na). Mp. 150–152°C. The powder X-ray diffraction results indicated an amorphous state (Figure 2). ^1^H-NMR (600 MHz, DMSO-*d*6): δ = 8.58 (1H, dd, *J* = 2.4, 0.6 Hz), 7.76 (1H, dd, *J* = 8.4, 2.4 Hz), 7.23 (1H, d, *J* = 8.4 Hz), 6.77 (1H, dd, *J* = 8.4, 2.2 Hz), 6.76 (1H, d, *J* = 2.2 Hz), 6.19 (1H, dd, *J*= 8.4, 0.6 Hz), 3.94 (2H, q, *J* = 6.9 Hz), 3.71 (2H, d, *J* = 6.0 Hz), 3.26 (1H, seq, *J* = 6.0 Hz), 2.03 (1H, seq, *J* = 6.6 Hz), 1.21 (6H, d, *J* = 6.6 Hz), 1.11 (3H, t, *J* = 6.9 Hz), 1.00 (6H, d, *J* = 6.6 Hz).

1. ^1^H NMR

NEt-3IB•Na

**Figure 1.**

1. **Elemental Analysis**

| Compd. |  | C (%) | H (%) | N (%) |
| --- | --- | --- | --- | --- |
| NEt-3IB・Na  2/3 H_2_O | Anald. | 64.62 | 7.08 | 7.09 |
|  | Calcd.^1)^ | 64.60 | 7.31 | 7.17 |

^¹)^ Calculated using ChemDraw version 22.2.0.

1. **Comparison of Melting Point**

| Compd. | Melting point (ºC) |
| --- | --- |
| NEt-3IB | 194–196 (Ref 2) |
| NEt-3IB •Na | 150–152 |

1. **Comparison of water solubility**

| Compd. | Solubility (PBS, pH 7.4, µM) | | |
| --- | --- | --- | --- |
|  | 2 h | 4 h | 24 h |
| NEt-3IB | 3.56 | 3.50 | 3.03 |
| NEt-3IB•H_2_O | 1.56 | 1.48 | 1.61 |
| NEt-3IB•Na | 5229.3 | 5246.3 | 4991.5 |

1. **Comparison of powder X-ray diffraction**

Blue: NEt-3IB

Red: NEt-3IB•Na

**Figure 2.**

**References**

1. *Chem Pharm Bull* (Tokyo). **2022**, *70(2)*, 146–154. doi: 10.1248/cpb.c21-00911.
2. *Chem Pharm Bull* (Tokyo). **2023**, *71(4)*, 282–288. doi: 10.1248/cpb.c22-00817.
